# Fibroepithelial polyp of palatine tonsil: a case report

**DOI:** 10.11604/pamj.2021.39.276.31057

**Published:** 2021-08-27

**Authors:** Katerina Marini, Konstantinos Garefis, James Philip Skliris, George Peltekis, Anna Astreinidou, Vasiliki Florou

**Affiliations:** 1 Department of Otorhinolaryngology Head and Neck Surgery, Gennimatas General Hospital, Thessaloniki, Greece,; 2 2^nd^ Academic ORL, Head and Neck Surgery Department, Aristotle University of Thessaloniki, Papageorgiou Hospital, Thessaloniki, Greece,; 3 Department of Pathology, Theageneion Cancer Hospital, Thessaloniki, Greece

**Keywords:** Fibroepithelial polyp, tonsil, surgery, case report

## Abstract

Fibroepithelial polyps represent a frequent cutaneous lesion of mesodermal origin, with a prevalence of 1.2% and are rarely located at palatine tonsils. We present a rare clinical report of a 70-year-old female patient with fibroepithelial polyp of palatine tonsil. This entity represents the eighth case of palatine tonsil fibroepithelial polyp in the English literature. She presented with a polypoid mass at the right tonsil and unspecified throat symptoms. Physicians should pay attention to such lesions because of the residual risk of malignant transformation, along with non-specific symptoms. Differential diagnosis was among neurofibroma, lipoma, squamous papilloma and fibroepithelial polyp. Histopathological examination following tonsillectomy showed a structure rich in vesicles inside lamina propria and surrounding inflammation, establishing the diagnosis of a fibroepithelial polyp. It requires vigilance during complete clinical examination, in order to detect masses at patients with throat symptoms that could have remained undiagnosed until they become even life threatening.

## Introduction

Fibroepithelial polyps (FPs) are benign lesions with exceptionally low incidence of malignant change [1]. They are frequently caused by hyperplasia of fibrous connective tissue [2]. Fibroepithelial polyps are of mesothelial origin and most commonly arising from the skin and rarer from the mucosa of the neck, face and trunk. There have been also described at the mucosa of the oral and nasal cavity, the oropharynx, and the hypopharynx. Alternatively, they are called fibromas or acrochordons [3]. There are various other unusual locations of FPs´ growth such as bronchi, genitals and ureteropelvic system [4-7]. However, very few reports describe palatine tonsils as the place of origin, even though a great variety of benign lesions arise from them [8]. This case report presents a patient with a large FP located at the right palatine tonsil, that was treated with tonsillectomy. Tumours like FP of palatine tonsil have a variety of symptoms, from no symptoms to life threatening. Although they have low incidence of malignant transformation, their location at the oropharynx makes them potentially dangerous, while when their dimensions increase they could cause airway obstruction. Our case report aims to offer an informative description, enrich the existing bibliography and contribute to clinical vigilance in the management of tumours of the oropharynx.

## Patient and observation

**Patient information:** a 70-year-old female patient presented to the ear, nose, and throat (ENT) outpatient department, mentioning a four-month history of throat lump. There was no history difficulty in swallowing and breathing, throat pain, change in voice or injury. It is of importance that the patient did not report foreign body sensation in her throat, ipsilateral to the lesion. According to her medical history, she suffered from hypertension and hyperlipidemia and was under medication. The patient did not mention a significant family history. She was a housekeeper, married with children and grandchildren and was under normal psychological condition. She did not mention any surgeries at the oropharynx or nasopharynx.

**Figure 1:**
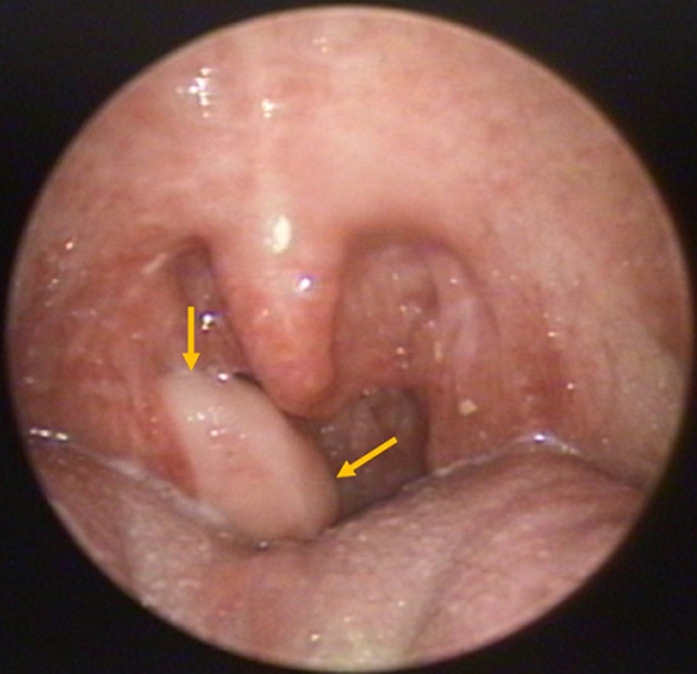
the fibroepithelial polyp (yellow arrows) arising from the superior pole of the right palatine tonsil

**Clinical findings:** the examination of the oropharynx revealed a polypoid, painless, well-defined and smooth-surfaced lesion arising from the superior pole of the right palatine tonsil, with dimensions of approximately 2.5 x 1.5cm (Figure 1). It was elastic in texture and was attached to the palatine mucosa through a stalk. Cervical lymph nodes were normal in size and unpalpable.

**Timeline of current episode:** the patient had a 4-month history of throat lump when she came to our hospital. She visited the emergency department of another hospital, about one month after the onset of the symptoms. She did not receive any diagnosis or treatment, so the throat lump insisted. She visited our outpatient department and one month later she underwent tonsillectomy.

**Diagnostic assessment:** pre-operative blood test results did not reveal any abnormal values, while no imaging examinations were necessary. Similarly, the rest of ENT examination was normal.

**Diagnosis:** fibroepithelial polyps was not the first diagnosis that we considered. We included tumours such as angiolipomas, lipomas, neurofibromas, lymphangiomas and squamous papilomas. Prognosis of FPs is excellent when they are completely removed, as they are benign lesions, while the relevant risk of malignancy is very low, about 0.37% [1].

**Figure 2:**
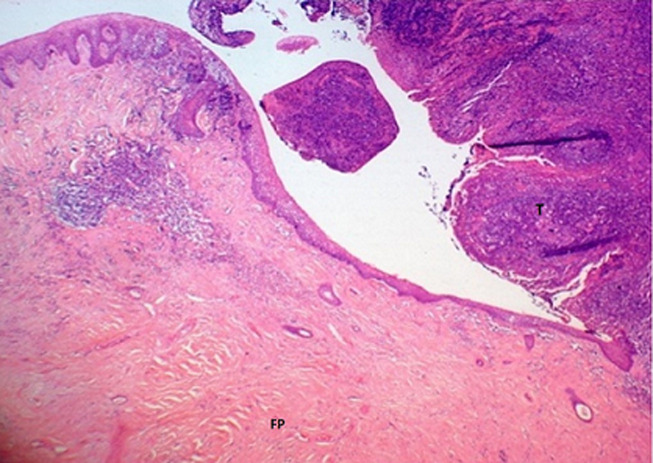
photomicrograph of the fibroepithelial polyp (FP); stratified squamous epithelium, vascular channels and perivascular infiltration of lymphocytes and plasma cells; the tonsil (T) is shown on the right (H and E, x25); H sand E indicates hematoxylin and eosin

**Therapeutic intervention:** a cold steel dissection bilateral tonsillectomy under general anesthesia was performed. Both tonsils, the right one containing the polypoid mass and the left, the normal one, were sent for histopathological examination. Results from the pathology department showed, in macroscopic description, a protruded, ash grey colored, polypoid lesion at the superior pole of the right tonsil. Microscopically, the subepithelial stroma was containing variable sized, thin walled, vascular channels, along with perivascular infiltration of lymphocytes and plasma cells. All these findings were compatible with a fibroepithelial polyp, which was completely removed (Figure 2). There was no evidence of dysplasia or malignancy.

**Follow-up and outcome of interventions:** postoperative care was uncomplicated, and the patient was discharged the second postoperative day in excellent general condition. It was decided that it was not necessary to carry out further examinations because of the benign nature of the mass and the complete excision of it. At 1-year follow-up period assessment, the patient was symptom-free and there was no evidence of FP recurrence.

**Patient perspective:** *“when I first visited your department, I was quite disappointed because I had a 4-month history of throat lump and could not find a solution to my problem. That symptom made me depressed because I thought that I would die with it. The fact that you detected the polyp, made me trust you and gain a positive attitude towards treatment. Although I thought that the treatment would be medical, I finally decided to proceed to surgical treatment. I was fully informed about the procedure, its duration, its risks and the recovery time. I was in excellent condition during my stay at the hospital, as well as during recovery. At my follow-up appointments, I was optimistic and happy and relieved from my throat symptoms”*.

**Informed consent:** patient was fully informed about the risks, benefits and alternatives of the proposed treatment. She was also informed about the case report and the fact that her case was rare. She gave the permission to write and publish a case report, in order to add some more information about such cases to the existing literature, and she provided a written informed consent.

## Discussion

Fibroepithelial polyps, also known as acrochordons or soft fibromas, are benign, mainly solitary cutaneous protrusions that only very rarely undergo malignant change [1]. They can be found at skin or mucous surfaces of the whole body. They are of mesodermal origin and the most frequent location is in the head and neck area (cheek, tongue, lips, pharynx), while age and sex have no association with their prevalence [2,3]. Age and sex have no association with prevalence, which is 12 cases per 1000 population with a slight male predominance [3,4]. To our knowledge, this case report represents the eighth patient with fibroepithelial polyp located at palatine tonsils in the English literature [8-14]. There are two prevailing theories regarding the formation of FPs: 1) hyperplasia of fibrous connective tissue and; 2) chronic inflammatory hyperplasia, out of which, the second is the weakest [2]. It is worth mentioning that an association between acrochordons and diabetes, hyperlipidemia and the medical components of metabolic syndrome, has been reported [15].

Most patients are asymptomatic, but polyps can sometimes cause cough, chocking, or foreign body sensation [10]. Moreover, symptoms and problems may occur when polyps are located at specific areas, for example they can cause occlusion when they are located inside bronchi [7]. Thus, when polyps are asymptomatic, their detection can become challenging and therefore they are underdiagnosed. Another reason why those lesions remain undetected is that they are similar to the normal mucosa of the pharyngeal area, so they cannot be detected easily if they do not cause symptoms. Although most cases of fibroepithelial polyps of tonsils have been detected in adults, some cases have been described in children too, but they are remarkably fewer. Symptoms, location, treatment and histopathological findings do not differ from adult patients [16]. Fibroepithelial polyps differential diagnosis includes lipomas, plasma cell granulomas, lymphangiomas, neurofibromas, schwannomas, squamous papillomas, fibromyxomas, hairy polyps or angiolipomas [10,11,13]. Concerning their management, surgical excision is the treatment of choice, as there is always the risk of malignant transformation or airway obstruction if the lesion is large enough to lead to the latter emergency situation [10].

## Conclusion

Fibroepithelial polyps constitute a very rare benign lesion when they arise from the palatine tonsils. When they do not cause obstructive phenomena, they are not life-threatening. Surgical excision is the treatment of choice, as there is always the risk of malignant transformation or airway obstruction. Despite not common they should be considered by clinicians in the differential diagnosis when such tumours are revealed at palatine tonsils during intraoral examination.
